# Characteristics of a CaSO_4_ composite oxygen carrier supported with an active material for *in situ* gasification chemical looping combustion of coal†

**DOI:** 10.1039/c8ra03425g

**Published:** 2018-06-27

**Authors:** Yongzhuo Liu, Minggang Gao, Xintao Zhang, Xiude Hu, Qingjie Guo

**Affiliations:** Key Laboratory of Clean Chemical Engineering of Colleges and Universities of Shandong Province, Qingdao University of Science and Technology Qingdao 266042 People's Republic of China qj_guo@yahoo.com; State Key Laboratory of Coal Clean Utilization and Ecological Chemical Engineering, Ningxia University Yinchuan 750021 People's Republic of China

## Abstract

CaSO_4_ is considered to be a potential oxygen carrier for chemical-looping combustion (CLC) due to its cheapness and high oxygen transport capacity. To improve the physicochemical stability of the CaSO_4_ oxygen carrier, CaSO_4_ composite oxygen carriers supported with clay, cement, and ash separately were prepared. It was found that the attrition resistance of the CaSO_4_ oxygen carrier composed of clay and cement improved due to the bond action of clay and cement. The reactivity of the composite oxygen carrier with coal was investigated in a thermogravimetric analyser (TGA) and fluidised bed. Sulphurous gas products were analysed by mass spectrometry (TG-MS) and gas chromatography (GC). Based on the catalysis of the active components in clay, cement and ash, the reaction rate of CaSO_4_ with coal was improved by the active materials. However, the side reaction generating the sulphurous gas was severe in both the reduction and oxidation stages, especially when using steam as the gasifying agent. To enhance the regeneration, the CaSO_4_/clay composite oxygen carrier was upgraded by adding CaO. It was demonstrated that SO_2_ release can be restrained in both the reduction and oxidation stages when the mass ratio of CaO to the CaSO_4_/clay composite oxygen carrier was higher than 1. At this point, the corresponding oxygen transport capacity was about 14.1 wt%.

## Abstract 2

The upgraded CaSO_4_/clay composite oxygen carrier possessed high physicochemical stability and an oxygen transport capacity of 14.1 wt%.

## Graphical abstract

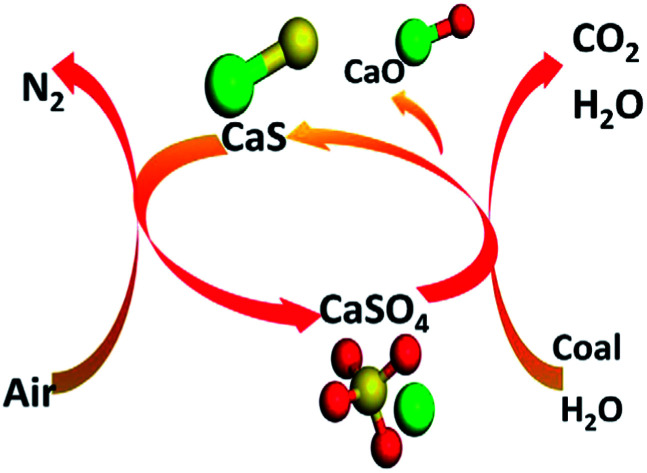

## 1. Introduction

It has been acknowledged that the emission of greenhouse gas (CO_2_), mainly caused by extensive utilization of fossil fuels, brings about global warming and a rise in sea level. To minimize CO_2_ emission in a short-medium term, CO_2_ capture and storage (CCS) technologies are practical options. Because of the inherent separation of CO_2_ without the need for extra energy, chemical-looping combustion (CLC) was identified as a promising technology for fossil fuel combustion.^1,2^ Oxygen carrier particles were used to divide the traditional combustion reaction into two-step reactions occurring separately in two interconnected reactors, which can avoid direct contact between air and fuel.

CLC can be applied for a variety of fuels including gaseous fuels, liquid fuels,^3,4^ and even solid fuels such as coal and biomass.^2,5^ CLC of coal is undergoing a high degree of development, since coal is expected to continue being one of the main energy sources in the medium term.^6^ To direct the chemical looping combustion of coal, *in situ* gasification chemical looping combustion (iG-CLC) has been proposed.^1,2^ Compared with CLC of gaseous fuels, iG-CLC of coal faces two challenges. On the one hand, the char gasification rate is the limiting step of reactions in the fuel reactor, which greatly determines the CO_2_ capture efficiency. To maximize the conversion of char in the fuel reactor, high temperature and a highly reactive oxygen carrier have been suggested.^7^ On the other hand, the effect of ash on the reactivity of oxygen carrier and its separation from the oxygen carrier have yet to be further explored. Oxygen carrier particles are prone to deactivation because of particle sintering and pore blocking by fine ash.^8^ Additionally, it is inevitable that some oxygen carrier particles will be extracted with the coal ashes when they are removed from the system. Ultimately, both challenges depend on the preparation of an appropriate oxygen carrier.

An alternative route, the so-called chemical looping with oxygen uncoupling (CLOU), has been proposed to improve iG-CLC performance.^9^ Oxygen carrier materials, such as synthetic materials of CuO and Mn_2_O_3_, with the capability to release gaseous oxygen in the fuel reactor have been used in CLOU. Excellent performance regarding combustion efficiency has been shown by these oxygen carriers.^10,11^

To realize the CLC process, it is important to find an inexpensive oxygen carrier available in large quantities and with sufficient oxygen transport capacity. The oxygen carrier should possess the ability to maintain excellent reactivity during many reduction/oxidation cycles and withstand mechanical handling without severe physical degradation or loss in performance.^12^ With respect to CLC of gaseous fuels, metal oxides such as Ni-based, Cu-based, Co-based, Fe-based, and Mn-based metal oxides and their metal blends have been regarded as oxygen carrier candidates and thus, they have been widely investigated. In the case of coal CLC, the inexpensive and widely available natural ore or by-products from industrial processes are promising options. Up to now, most studies have focused on iron-based materials from different sources such as ilmenite.^13,14^ In these cases, complete combustion has not been achieved, and unconverted products such as H_2_, CO and CH_4_ exit the fuel reactor with the CO_2_ stream. Furthermore, complete combustion has still not been achieved using more reactive materials such as iron ore or the waste Fe-enriched sand fraction from alumina production.^15–17^

In addition to these materials, CaSO_4_ is deemed to be a promising oxygen carrier due to its abundance, low cost and high oxygen transport capacity; an oxygen transport capacity of 47 wt% has been reported, which is higher than those of any other proposed materials.^18^ The reactivity and kinetics of CaSO_4_ as the oxygen carrier in CLC for both gases (CO and CH_4_) and coal have been extensively investigated.^19–24^ Moreover, the feasibility and characteristics of the CaSO_4_ oxygen carrier for coal chemical looping gasification (CLG) have also been demonstrated.^25–27^ Based on the desulphurization of limestone in a circulating fluidised bed combustion boiler, Alstom proposed a limestone chemical looping combustion process (LCL-C) in which CaCO_3_ was continuously fed together with coal to produce CaSO_4_ as an oxygen carrier *via* sulphur retention.^28^ Subsequently, Alstom demonstrated good performance of LCL-C regarding CO_2_ capture in a 3 MWth CLC unit.^29^ However, there is no detailed information available in the literature regarding the LCL-C process. Recently, Abad *et al.*^30^ verified the LCL-C process with CO_2_ capture and *in situ* desulphurization using mass and energy balances, and they concluded that the oxygen transport capacity of sulphated limestone was about 16.7 wt%, corresponding to a fraction of 23 wt% CaSO_4_. However, the CaSO_4_ oxygen carrier formed *via* continuous sulphur retention of CaCO_3_ can intensify the effect of coal ash on the oxygen carrier and reduce the lifespan of the oxygen carrier.

The main chemical reactions involved in the iG-CLC process using CaSO_4_ as the oxygen carrier are listed in (R1)–(R11). In the fuel reactor, the reactions (R1)–(R3) are the desired reactions, whereas (R4)–(R8) are the main side reactions generating sulphurous gas, which diminish the oxygen transport capacity of CaSO_4_. In the air reactor, (R10) is our desired reaction, whereas (R9) and (R11) are the main side reactions generating sulphurous gas.

R1 CaSO_4_ + 2C → CaS + 2CO_2_, Δ*H*_298_ = 175.61 kJ mol^−1^

R2 CaSO_4_ + 4CO → CaS + 4CO_2_, Δ*H*_298_ = −169.23 kJ mol^−1^

R3 CaSO_4_ + 4H_2_ → CaS + 4H_2_O, Δ*H*_298_ = −98.04 kJ mol^−1^

R4 CaSO_4_ + CO → CaO + 4CO_2_ + SO_2_, Δ*H*_298_ = 222.93 kJ mol^−1^

R5 CaSO_4_ + 4CO → CaO + 3CO_2_ + COS, Δ*H*_298_ = −77.35 kJ mol^−1^

R6 CaSO_4_ + H_2_ → CaO + H_2_O + SO_2_, Δ*H*_298_ = 264.20 kJ mol^−1^

R7 CaSO_4_ + 4H_2_ → CaO + 3H_2_O + H_2_S, Δ*H*_298_ = 57.58 kJ mol^−1^

R8 CaSO_4_ → CaO + SO_2_ + 1/2O_2_, Δ*H*_298_ = 50.30 kJ mol^−1^

R9 CaSO_4_ + CaS → 4CaO + 4SO_2_, Δ*H*_298_ = 1060.94 kJ mol^−1^

R10 CaS + O_2_ → 4CaSO_4_, Δ*H*_298_ = −959.51 kJ mol^−1^

R11 CaS + 1/2O_2_ → 4CaO + SO_2_, Δ*H*_298_ = −458.53 kJ mol^−1^

The main drawback of the CaSO_4_ oxygen carrier is its low reactivity compared to other materials and the release of SO_2_ at temperatures of interest for CLC. Temperature greatly affects side reactions.^18–22^ Song *et al.*^19^ observed that CaS was the dominant compound at 900 °C, whereas the presence of CaO was more relevant at 950 °C in the fuel reactor. Shen *et al.*^31^ proposed 900–950 °C and 1050–1150 °C as the suitable ranges of temperatures for the fuel and air reactors, respectively. The CaSO_4_–CaS–CaO phase diagram predicts that CaS is stable at the high partial pressure of sulphurous gas and reducing gas H_2_/CO using gaseous fuel.^32,33^ Tian *et al.*^33^ observed that the solid product was almost pure CaS when the partial pressure of CO or H_2_ was higher than 40 kPa. However, the side reactions generating sulphurous gas are unavoidable in the fuel reactor due to the aim of minimizing the unconverted products such as H_2_, CO and CH_4_. Especially, ashes and a complex atmosphere with coal as the fuel exert tremendous influence on the side reactions emitting sulphurous gas. In addition, sulphur evolution is also a problem in the air reactor. The evolution of SO_2_ can also occur *via* undesired reactions (R9) and (R11).

To improve the reactivity of CaSO_4_ and avoid the generation of SO_2_ from CaSO_4_ in both reactors, several alternatives have been proposed. Some studies have reported that the addition of Fe_2_O_3_ to CaSO_4_ by different preparation methods^34–37^ may help inhibit undesired side reactions and improve the reactivity of CaSO_4_. Other authors have suggested introducing CaO or CaCO_3_ together with CaSO_4_ (ref. 33) to capture SO_2_ released during CaSO_4_ decomposition and inhibit the aggregation of CaSO_4_ oxygen carrier particles at high temperature. Other studies have reported that the addition of K_2_CO_3_*via* the impregnation method can improve the reactivity between the oxygen carrier and coal.^38,39^

In open literature, the investigated CaSO_4_ oxygen carriers are mostly pure CaSO_4_, anhydrite ore having high CaSO_4_ content (>94 wt%) or CaSO_4_ modified by adding a small amount of active metal oxide.^34–39^ However, the conversion between the oxidized state (CaSO_4_) and the reduced state (CaS) may significantly change the structure of the oxygen carrier due to its high oxygen transport capacity. On the other hand, a large amount of heat generated by the oxide reaction of CaS can result in an increase in the localized temperature of the particles and eventually sintering of the oxygen carrier. As a consequence, the physicochemical stability of the CaSO_4_ oxygen carrier can be degraded. The addition of bulk support materials such as Al_2_O_3_, SiO_2_, and TiO_2_ into the oxygen carrier can be an alternative approach to improve its physicochemical stability. Besides, the attrition resistance of the CaSO_4_ oxygen carrier has been seldom investigated. Cabello *et al.*^40^ evaluated the attrition resistance of 23 oxygen carriers and concluded that the oxygen carrier with a crushing strength higher than 1 N and an Air Jet Index (AJI) lower than 5% is eligible for the CLC process.

To improve the physicochemical stability and to avoid the release of SO_2_ from the CaSO_4_ oxygen carrier, three active support materials, *i.e.*, clay, cement and ash were employed in this study. The attrition resistance and the reactivity between the CaSO_4_ composite oxygen carrier and coal were evaluated. Meanwhile, the generation of sulphurous gas during reduction and oxidation was analysed by TG-MS. Finally, the improvement in the CaSO_4_/clay composite oxygen carrier through the addition of CaO was investigated.

## 2. Experimental section

### 2.1 Materials and characterization

The chemical composition analysis by XRF of the used clay, fly ash and cement is summarized in Table 1. These active support materials mainly contained inert supports such as Al_2_O_3_, SiO_2_, catalysts for coal gasification, such as potassium and sodium salts, and the sulphur adsorption agent CaO. SiO_2_ was selected for comparison. It was demonstrated that the main component of clay and fly ash was SiO_2_ and that of cement was CaO. According to the pre-experiment, the mass ratio of CaSO_4_ and support materials (clay, fly ash, cement and SiO_2_) was 3 : 2. The corresponding oxygen transport capacity was about 28.2 wt%. The composite oxygen carriers were prepared by the mechanical mixing method. First, CaSO_4_ (ACS reagent) and the support material were obtained by passing through a 200-mesh sieve separately. CaSO_4_ and each support material having the desired mass ratios were placed in the beaker and stirred for 30 min. Then, enough deionized water was added into the beaker until the mixed sample was immersed in water. Subsequently, the mixed sample was stirred for 20 min and dried overnight. The dried samples were calcined at 800 °C for 4 hours in a muffle furnace. Then, for further use, the calcined oxygen carrier was crushed and sieved to obtain the determined particle size, *i.e.*, 98–180 μm. The proximate and ultimate analysis of the used coal is displayed in Table 2.

**Table 1:** Component analysis of clay, fly ash and cement

materials	SiO_2_, wt%	Al_2_O_3_, wt%	Fe_2_O_3_, wt%	CaO, wt%	Others, wt%
Clay	57.98	7.05	4.57	8.52	21.88
Fly ash	57.61	9.86	6.19	17.8	8.54
Cement	20.93	4.55	1.05	64.34	9.13

Proximate and ultimate analysis of used coal

**Table:** 

Proximate analysis/wt%, ad			
M	V	A	FC
8.30	29.46	10.21	52.03

**Table:** 

Ultimate analysis/wt%, ad				
C	H	O	N	S
65.00	3.83	11.38	0.88	0.4

The microstructures of the oxygen carrier particles were observed by SEM (scanning electron microscope, JEOL JSM-6300). The element distribution in the solid particles was analysed by EDX (Energy-Dispersive X-ray Microanalyser, JEOL JED-2100). The crystalline phase of the fresh oxygen carrier was analysed using an X-ray diffractometer (Rigaku D/MAX-2500, Japan), with 2*θ* ranging from 10° to 90° with a step of 0.02° s^−1^. All X-ray diffraction (XRD) patterns were analysed using Jade 6.0 of Material Data, Inc. (MDI).

The reactivities between coal and composite oxygen carriers were studied using a thermogravimetric analyser (TGA, Netzsch STA 409 PC, Germany). The desired mass ratio of the composite oxygen carrier to coal was determined by the fixed carbon in coal and by the transfer of oxygen in the composite oxygen carriers, according to the reaction CaSO_4_ + 2C → CaS + CO_2_. To start the experiment using TGA, about 12 mg of the desired mass ratio mixture was put into the crucible and heated directly from ambient temperature to 900 °C at a heating rate of 30 °C min^−1^ with N_2_ as a purge gas, with or without H_2_O as a gasifying gas. The flow rates of N_2_ were determined to be approximately 20 ml min^−1^, whereas that of H_2_O was determined by its saturated vapor pressure in N_2_ at 30 °C for TGA. The temperature was maintained at 900 °C for 40 min and then elevated to 1000 °C. At the same time, the purge gas was switched to O_2_ to oxidize the oxygen carrier for 20 min.

To investigate the release behavior of sulphurous gas during the redox reaction, the sulphurous gas was analysed using TG-MS (STA449F1-QMS 403 D, Netzsch, Germany). To protect the device, the starting temperature was set as 150 °C with a heating rate of 15 °C min^−1^, and the oxidation stage was conducted at 950 °C. The flow rate of water was determined to be 0.2 g min^−1^. The other conditions were the same as the conditions of the former TGA analysis (Netzsch STA 409 PC, Germany).

### 2.2 Experimental setup and procedure

**Fig. 1:**
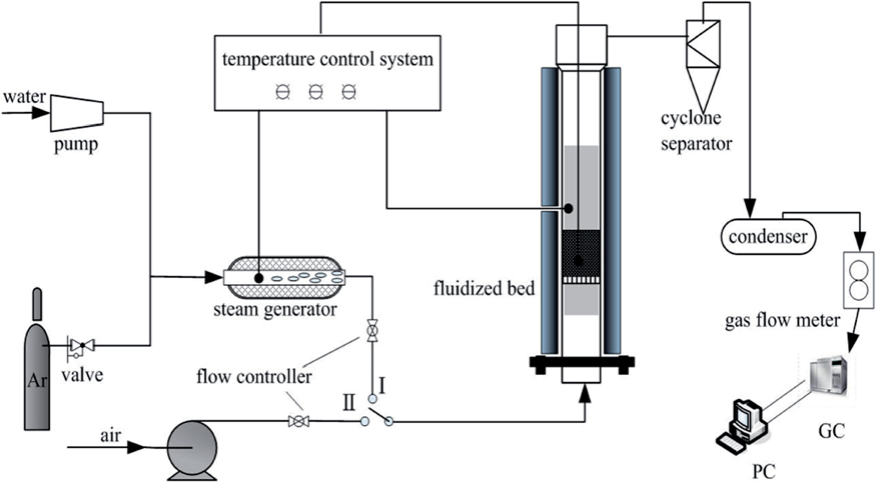
Schematic diagram of the experimental fluidised-bed setup.

The schematic diagram of the fluidised-bed setup used in the experiments is presented in Fig. 1. The CLC system consisted of a high-temperature fluidised bed (stainless steel, *Φ*50 × 650 mm), temperature control unit, gas feeding system (steam generator, gas cylinders, and gas flow meter), gas analysis system, and data acquisition system. The reactor was electrically heated with a furnace. One K-type thermocouple was fixed in the section between the tube and the heater, and the other was fixed inside the tube.

To set up the experiment, a mixture of coal (4 g) and composite oxygen carrier (50 g) particles was fed into the fluidised bed in advance. The mass ratio was determined to keep the oxygen carrier in excess, which has been used in previously reported methods. During the reduction process, argon (0.4 L min^−1^) and steam (0.5 g min^−1^) were used as the purge gas and gasifying agent, respectively. After the temperature reached the given reduction temperature (900 °C), steam was introduced from the bottom of the reactor. The reduction reaction lasted for 40 min, during which coal was mostly converted into CO_2_ and water vapour. Gaseous products were sampled by employing gas bags every 2 min for analysing by gas chromatograph. After the reduction reaction was complete, the inlet gas was switched to air (0.2 L min^−1^) to oxidize the oxygen carrier for 1 hour. Subsequently, oxygen carrier particles were cooled to ambient temperature in an argon atmosphere and then collected for further analysis.

The gas in the gas bag was analysed directly by a gas chromatograph analyser (PE Clarus 500) using a TDX-01 packed column/thermal conductivity detector for syngas and a Propark QS packed column/flame photometric detector for sulphurous gas.

### 2.3 Data evaluation

(1) The gas concentration, *C*_i_, is the ratio of the volume of the exit gases (i = CO_2_, CO, CH_4_, H_2_, SO_2_) to total volume and is given by the following formula:
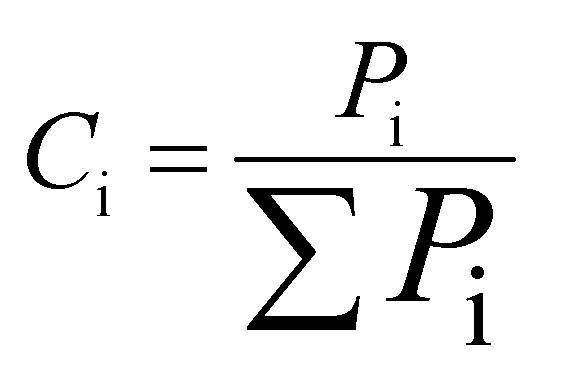


(2) The carbon conversion efficiency, *X*_c_, is the ratio of the carbon mole of carbonaceous gases (CO, CO_2_, and CH_4_) and the total carbon mass contained in the coal, and it is calculated as follows:
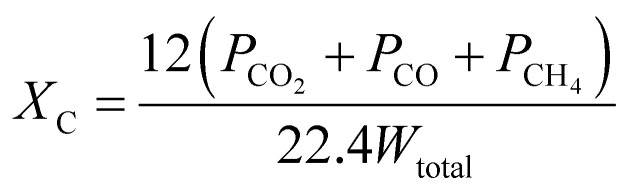


(3) The CO_2_ generation rate, *R*_CO2_, is calculated as follows:
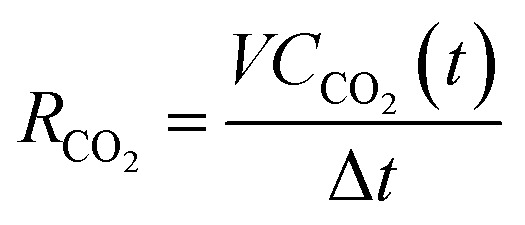


Here, *V* is the volume of generated gas in the time interval Δ*t*.

## 3. Results and discussion

### 3.1 Attrition performance

**Fig. 2:**
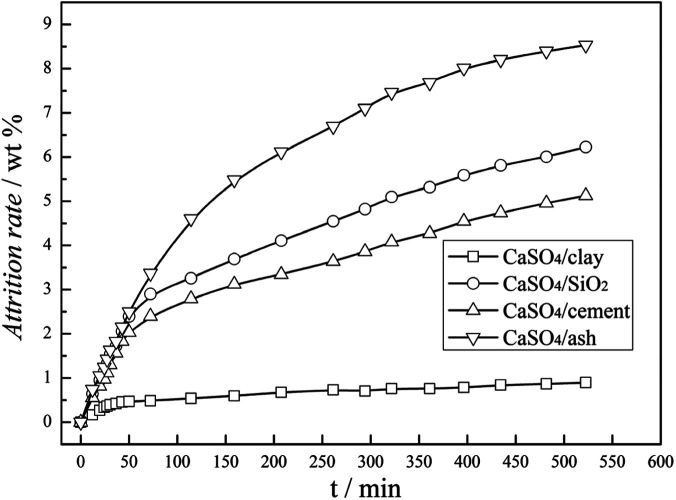
Attrition rate of CaSO_4_ composite oxygen carrier *versus* time.

The attrition resistance of three kinds of fresh composite oxygen carriers was determined using an attrition test system, configured according to the ASTM-D-5757 standard. As specified in the ASTM method, fifty grams particles was used for each sample tested. The samples were tested under 10 L min^−1^ of air flow, and the weight loss of fines was recorded at 1 and 5 h of time on stream. Particles with a size lower than 20 μm were considered as fines. The attrition rate was defined as the mass ratio of the accumulated fine particles to the total addition of oxygen carrier particles. As illustrated in Fig. 2, the attrition rate curves of three types of composite oxygen carriers *versus* time can be characterized by two stages: the rapid loss stage and the slow loss stage. The rapid loss stage was named as the fragmentation stage, which was mainly due to the surface asperities of the oxygen carrier particles, whereas the slow loss stage was named the abrasion stage, which reflected the attrition behaviour of the oxygen carriers for long-time operation in a fluidised bed. After a short time of the fragmentation stage, within 50 min, three kinds of composite oxygen carriers underwent the abrasion stage. It was demonstrated that the attrition rates of the CaSO_4_/clay and CaSO_4_/cement composite oxygen carriers were 1% and 5% within 500 min, respectively; thus they possessed excellent attrition resistance compared to CaSO_4_/SiO_2_. This resulted from the good bond action of cement and clay. On the contrary, the attrition rate of the CaSO_4_/ash composite oxygen carrier was 8.5% within 500 min, showing high risk of particle integrity failure. However, it should be stressed that further study in the continuous CLC pilot plant is necessary to determine whether the attrition resistance of the oxygen carrier is suitable for the CLC process.

### 3.2 Characterization of fresh composite oxygen carriers

**Fig. 3:**
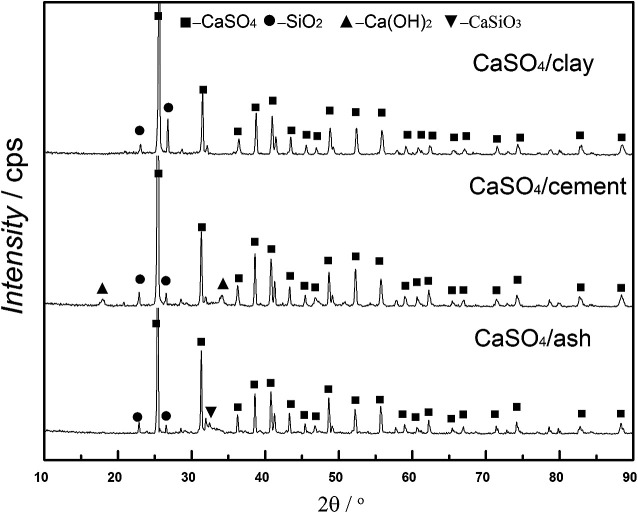
XRD patterns of fresh composite oxygen carrier.

XRD patterns of three kinds of fresh CaSO_4_ composite oxygen carriers are shown in Fig. 3, which clearly demonstrates that the main crystalline phase in the fresh oxygen carrier was CaSO_4_. The main component SiO_2_ in the clay and fly ash was observed in both the CaSO_4_/clay and CaSO_4_/ash composite oxygen carriers, whereas Ca(OH)_2_ was observed in the CaSO_4_/cement oxygen carrier. This was ascribed to the adsorption of water steam by CaO in the cement. Except for CaSiO_3_ in the CaSO_4_/ash composite oxygen carrier, no new phases in the three types of composite oxygen carriers were observed after calcinations; this indicated that CaSO_4_ did not significantly react with any component of clay, cement and ash. Al_2_O_3_ and Fe_2_O_3_ were not observed even though Fe and Al were present in clay, cement and ash. It should be pointed out that the peak corresponding to high content of CaSO_4_ might overlap or cover the Al_2_O_3_ and Fe_2_O_3_ peaks because of their relatively lower contents.

### 3.3 Reactivity of composite oxygen carriers with coal

**Fig. 4:**
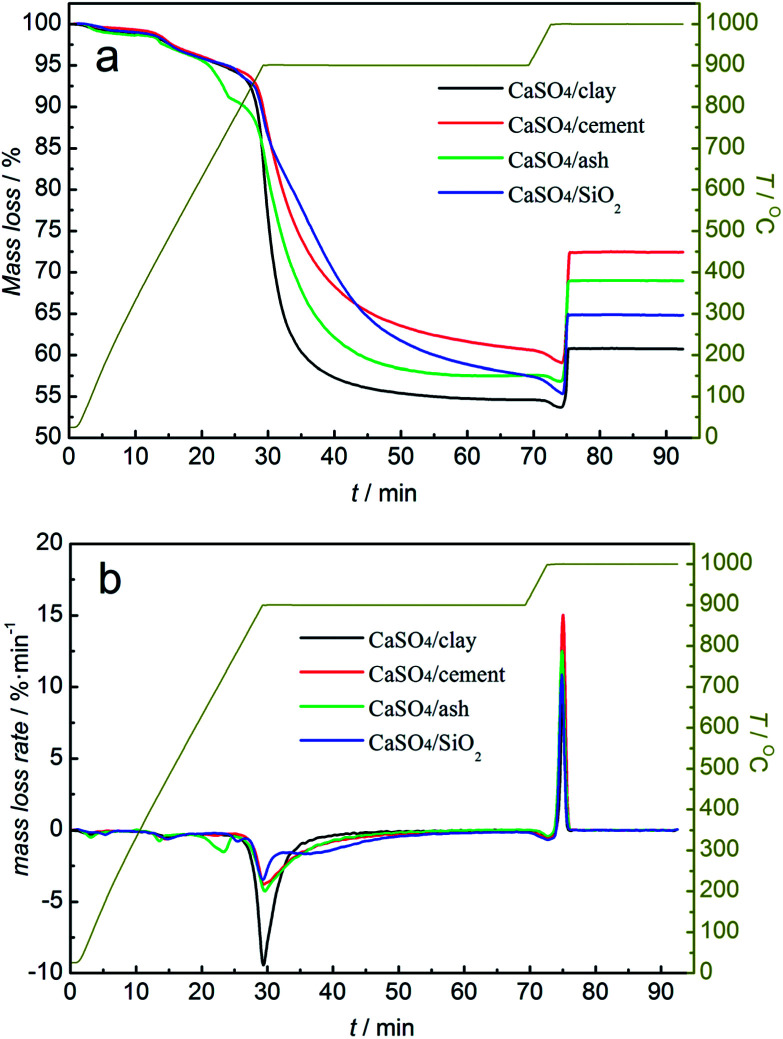
TG (a) and DTG (b) curves of composition oxygen carriers undergoing one redox cycle with coal without a gasifying agent and air, alternately.

Investigations into the reactivity of composite oxygen carriers with coal were carried out using TGA. The mass loss (TG) and the mass loss rate (DTG) of the mixture of the composite oxygen carrier and coal with the increasing temperature are demonstrated in Fig. 4. It was clearly found that all three composite oxygen carriers showed the same variation trend with the increasing temperature. With N_2_ as a purge gas, the mass loss at 100 °C was due to the drying of the mixture of the composite oxygen carrier and coal. The mass loss at 400 °C was caused mainly by coal pyrolysis. When the temperature rose to 850 °C, the mass of the mixed samples began to decline rapidly. The temperature was maintained at 900 °C for 40 min until the mass of the mixed samples began to level off. When the temperature was elevated to 1000 °C, the purge gas was switched to O_2_. The mass of the mixed samples first decreased and then increased instantly to a steady value.

In the reduction stage, as was depicted in Fig. 4b, the mass loss rate of the mixture of composite oxygen carriers with coal was higher than that of CaSO_4_/SiO_2_; this indicated that the addition of clay, cement and ash could significantly improve the reaction rate between CaSO_4_ and coal. The CaSO_4_/clay composite oxygen carriers had the highest reaction rate with coal. However, the mass losses of the three composite oxygen carriers differed from each other. The mass loss values were 45.38%, 39.35% and 42.49%, for CaSO_4_/clay CaSO_4_/cement and CaSO_4_/ash, respectively. One reason for this observation was that the mass rate of CaSO_4_ to active support materials changed during the preparation process, due to which the theoretical oxygen transport capacities of the composite oxygen carriers were different from each other. Another important reason was the generation of CaO from the side reaction, and its mass loss was different from that of CaS generated from the desired reaction. The occurrence of the side reaction was verified by the analysis of the generated sulphurous gas. The analysis of relative contents of the generated sulphurous gas with the increasing temperature using TG-MS will be discussed later.

In the oxidation stage, a mass decrease was observed in the mixed sample when the purge gas was switched to O_2_; this indicated that a small amount of coal did not react with the oxygen carrier, because the solid products generated during the reactions prevented the further reaction between the oxygen carrier and coal. The significant mass gain within 2 min was due to the oxidation reaction of CaS. However, the mass gain values were 7.11%, 13.37% and 12.14% for CaSO_4_/clay, CaSO_4_/cement and CaSO_4_/ash, respectively; all of these values were lower than the theoretical value (23.4%). Generally, the difference between the mass gain value and the theoretical value is derived from the generation of CaO in one redox cycle. When the mass sum of CaSO_4_ and CaO was 100% for the CaSO_4_/clay composite oxygen carrier, CaSO_4_ accounted for 79.64%, whereas CaO accounted for 20.36% after one redox cycle; in other words, 38.3% of CaSO_4_ was deactivated after one redox cycle. Similarly, it was calculated that 25.82% and 28.47% of CaSO_4_ in the CaSO_4_/cement and CaSO_4_/ash composite oxygen carriers were deactivated after one redox cycle. Among the three types of composite oxygen carriers, the mass gain percent of CaSO_4_/cement was the highest, whereas that of CaSO_4_/clay was the lowest; this can be explained by the effect of different active components on the side reaction of CaSO_4_. Thermodynamically, the components SiO_2_, Al_2_O_3_, and Fe_2_O_3_ were harmful to the regeneration of CaSO_4_ due to the formation of a eutectic mixture such as CaSiO_3_, CaAl_3_O_7_, CaAl_2_O_4_ and CaFe_2_O_4_. On the other hand, the component CaO was favourable to the regeneration of CaSO_4_ (please refer the supplement material). The higher content of CaO in cement gave rise to better regeneration of the CaSO_4_/cement composite oxygen carrier.

**Fig. 5:**
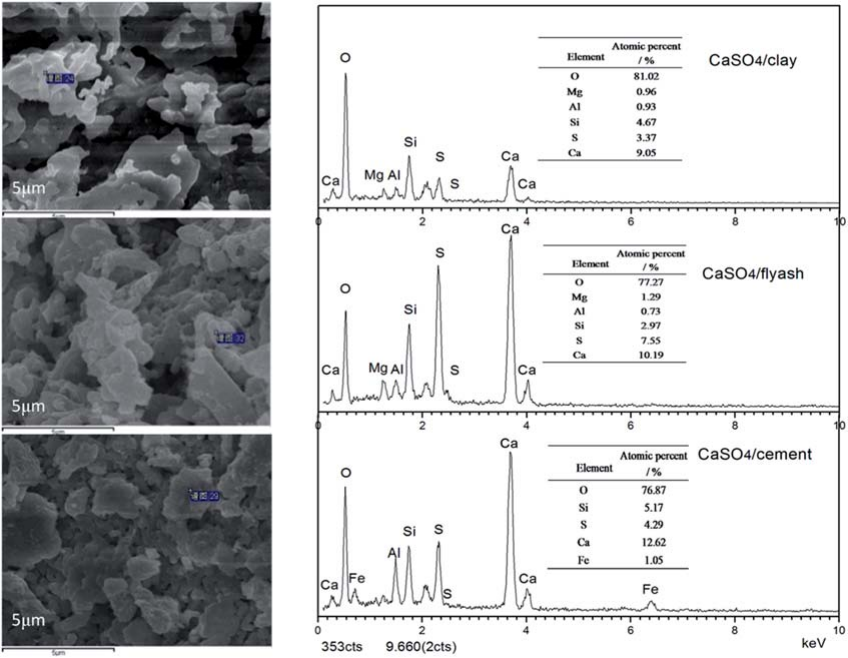
SEM-EDS images of solid products after one reduction with coal and one oxidation with air, alternately.

Solid products derived from composite oxygen carriers after one reduction with coal and one oxidation with air were analysed by SEM-EDS. As depicted by SEM in Fig. 5, no clear sintering was observed on the surface of the three composite oxygen carriers after one redox cycle; this was mostly due to the addition of active support materials. As shown by EDS analysis, the atomic percent of the sulphurous element in CaSO_4_ decreased relative to the calcium element. The mole ratios of Ca to S were 2.69, 2.94 and 1.35 for the CaSO_4_/clay, CaSO_4_/cement and CaSO_4_/ash composite oxygen carriers, respectively. Theoretically, the mole ratio of Ca to S is equal to 1, considering that both Ca and S are obtained from CaSO_4_. The mole ratio of Ca to S in the CaSO_4_/ash composite oxygen carrier was 1.35, indicating that the sulphur release due to the side reaction was lower; this result was in accordance with the aforementioned TG results, where the mass gain value was high. Nevertheless, due to the high content of Ca in cement, the theoretical mole ratio of Ca to S in the CaSO_4_/cement oxygen carrier was higher than that in CaSO_4_/clay. Therefore, the relative mass loss of the sulphurous element of CaSO_4_/clay was highest, though the Ca/S mole ratio was relatively low; this result was also in accordance with the TG results, where the mass gain percent of CaSO_4_/cement was highest, whereas that of CaSO_4_/clay was lowest. Clearly, sulphur loss in the three composite oxygen carriers occurred due to side reactions. It should be pointed out that sulphur release from coal was not taken into account.

**Fig. 6:**
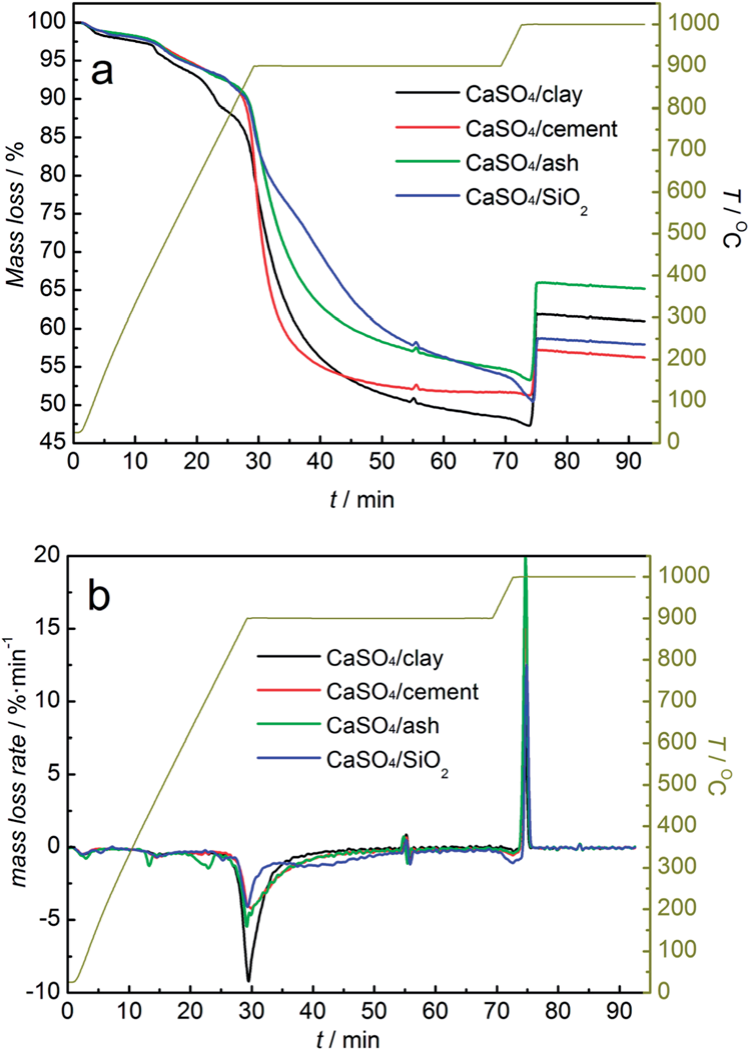
TG (a) and DTG (b) curves of composite oxygen carriers undergoing one redox cycle with coal using steam as the gasifying agent and air, alternately.

Redox reactions between the three types of composite oxygen carriers and coal with steam as the gasifying agent were investigated. The mass loss curve of the mixture of composite oxygen carriers and coal is shown in Fig. 6. As depicted in Fig. 6b, the mass loss rates of all the three types of composite oxygen carriers were accelerated at the reduction stage. The reaction rates of the three types of composite oxygen carriers with coal were clearly higher than that of the CaSO_4_/SiO_2_ oxygen carrier; this was ascribed to the higher gas–solid reaction rate between the gasified gas and the oxygen carrier compared to the solid–solid reaction rate between coal and the oxygen carrier. The coal gasification reaction generating gasified gases (H_2_ and CO) could be catalysed by the active components Fe_2_O_3_, CaO, potassium salt and sodium salt contained in clay, ash and cement. However, there was a slow decline in the mass of the mixed samples after a rapid decline, which was different from that without gasifying agent. According to our previous research,^41^ the reaction between CaSO_4_ and coal with steam as the gasifying agent transformed from reaction control to diffusion control. Diffusion control deteriorated the subsequent reaction between the composite oxygen carriers and coal or gasified gas. The mass loss values were 51.39%, 48.35% and 45.15%, for CaSO_4_/clay CaSO_4_/cement and CaSO_4_/ash, respectively, which were higher than that without gasifying agent.

At the oxidation stage, similar to the observations under inert atmosphere, when the purge gas was switched to O_2_ at 1000 °C, the mass of the mixed samples slightly decreased before increasing. It was demonstrated that the extent of the reaction of coal was still limited even after using steam as the gasifying agent. The mass gain values were 14.50%, 5.9% and 12.53% for CaSO_4_/clay, CaSO_4_/cement and CaSO_4_/ash, respectively, which were lower than the theoretical value (23.4%). Side reactions generating sulphurous gas occurred in the redox reactions of the three types of composite oxygen carriers with coal. It was also calculated that 23.32%, 40.47% and 27.63% of CaSO_4_ were deactivated in the CaSO_4_/clay, CaSO_4_/cement and CaSO_4_/ash composite oxygen carriers, respectively, after one redox cycle. Surprisingly, the mass gain percent of CaSO_4_/clay increased from 7.11% without gasifying agent to 14.50% with steam as the gasifying agent, whereas the mass gain percent of CaSO_4_/cement decreased from 13.37% to 5.90%. It can be inferred that the inhibition of CaO by the side reaction was greatly affected by steam. The mass gain percent of CaSO_4_/ash was 12.53%, and it was similar to that without gasifying agent. The positive effect of coal ash on the redox reaction of the CaSO_4_ oxygen carrier was noted. However, further investigation into the CaSO_4_/ash composite oxygen carrier was not taken into account due to the poor attrition resistance.

### 3.4 Release of sulphurous gas from CaSO_4_/clay and CaSO_4_/clay reacted with coal

To study the release behaviour of sulphurous gas during the redox reaction, the redox reactions of the CaSO_4_/clay and CaSO_4_/cement composite oxygen carriers with/without a gasifying agent were analysed by TG-MS. The potential sulphurous gas release included SO_2_ and COS without gasifying agent and SO_2_ and H_2_S with steam as the gasifying agent. The mass-to-charge ratios of SO_2_, COS and H_2_S were represented by 64, 60, and 34, respectively, in MS. The relative amount of sulphurous gas was considered to be proportional to ionic strength.

**Fig. 7:**
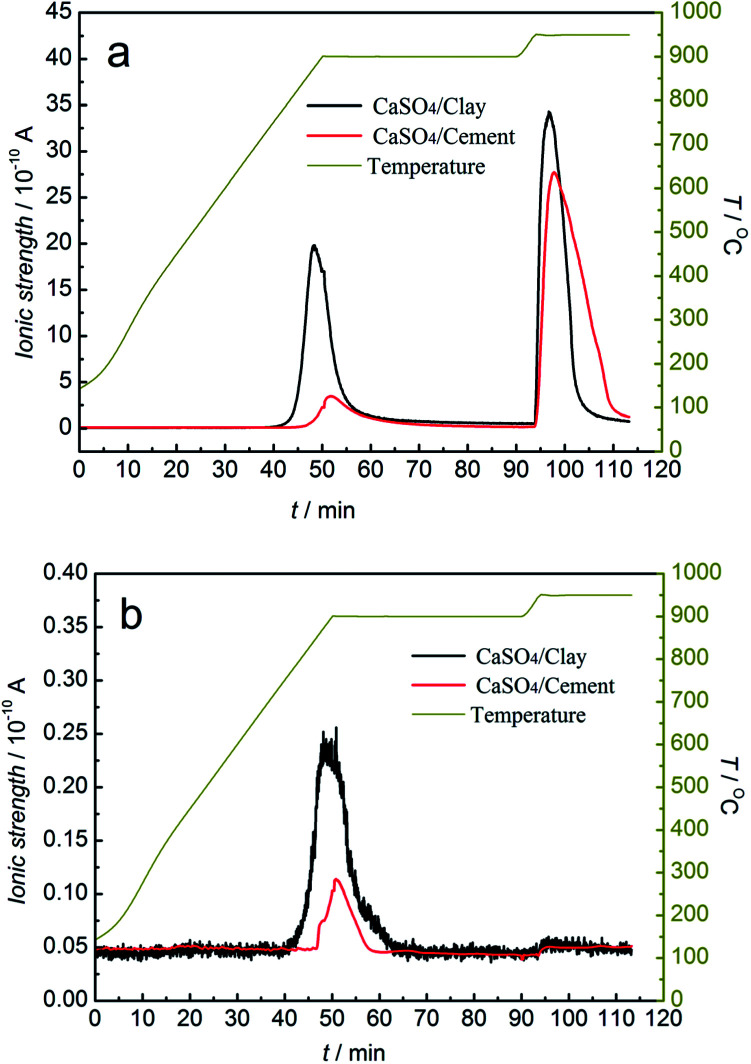
Mass spectrometric analysis of sulphurous gas products from TGA without the gasifying agent (a) SO_2_; (b) COS.

MS analysis of sulphurous gas products from TGA *versus* time and temperature without gasifying agent is depicted in Fig. 7. The generation of SO_2_ occurred in both the reduction and oxidation stages, whereas only a small amount of COS was released in the reduction stage. In the reduction stage, the generation of SO_2_ started from 778 °C and 840 °C for the CaSO_4_/clay and CaSO_4_/cement composite oxygen carriers, respectively. The release amount of SO_2_ with the CaSO_4_/cement composite oxygen carrier was far less than that with the CaSO_4_/clay composite oxygen carrier; this indicated that by restraining the side reactions (R4) and (R6), the CaSO_4_/cement composite oxygen carriers exhibited relatively high suppression ability of SO_2_ release, mainly due to the high content of CaO in cement. It should be noted that the sulphur present in coal was also released at the same time in the form of SO_2_ or COS. Besides, as shown in Fig. 7b, the initial temperature generating COS was relatively low, which was ascribed to the side reaction (R5).

In the oxidation stage, the release of SO_2_ was very high for both the CaSO_4_/clay and CaSO_4_/cement composite oxygen carriers. The SO_2_ peak area was larger for the CaSO_4_/cement composite oxygen carrier. The release of SO_2_ was most likely due to low oxygen partial pressure or the reaction of the generated CaSO_4_ with CaS, according to reaction equations (R11) and (R9), respectively.

**Fig. 8:**
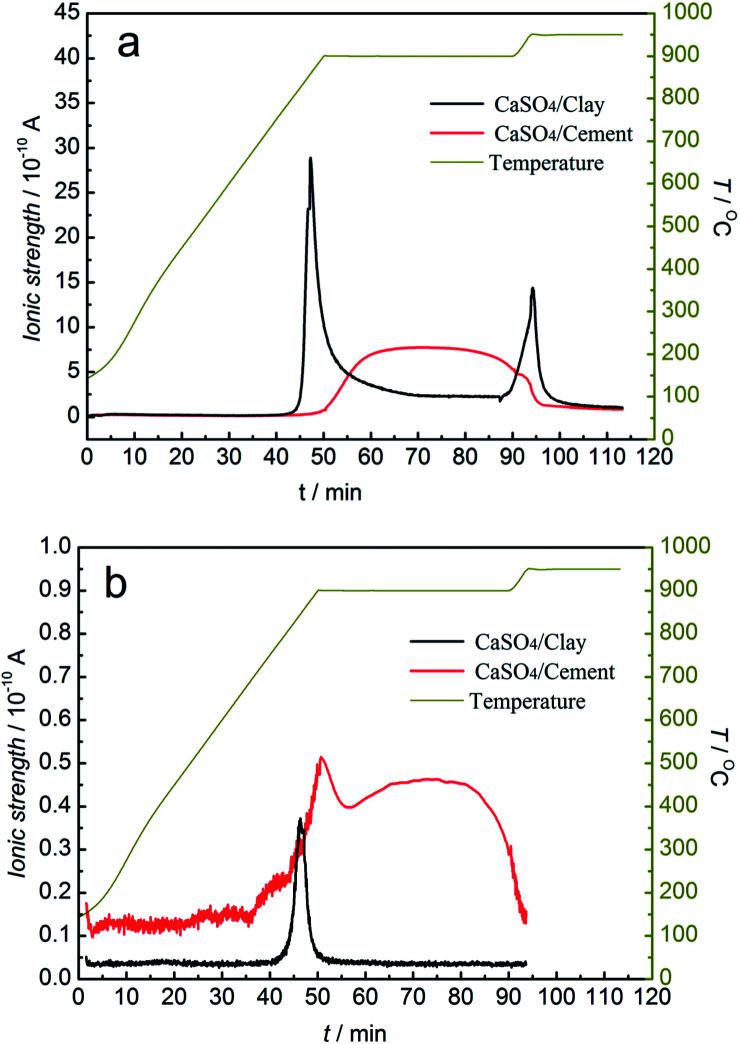
Mass spectrometric analysis of sulphurous gas products from TGA with steam as the gasifying agent. (a) SO_2_; (b) H_2_S.

As illustrated in Fig. 8, the SO_2_ release occurred in both the reduction and oxidation stages, whereas the H_2_S release occurred in the reduction stage using steam as the gasifying agent. The release amounts of SO_2_ for both composite oxygen carriers were greater than that under the inert atmosphere. At the same time, the release behaviour lasted for the entire reduction stage. Especially for CaSO_4_/cement, the release amount of SO_2_ and H_2_S was always high during the entire reduction stage, and it was higher than that for the CaSO_4_/clay composite oxygen carrier. This resulted in lower mass gain at the oxidation stage, as seen in Fig. 6. With steam as the gasifying agent, the gasification reaction of coal generated syngas (CO and H_2_). Thus, the generated syngas enhanced the side reactions (R4) and (R6) for releasing SO_2_ and the side reaction (R7) for releasing H_2_S. Considering the poor regeneration of CaSO_4_/cement with steam as the gasifying agent, it seemed that the steam reduced the suppression of sulphurous gas by CaO; this could be due to the effect of catalysis of other components in cement on the side reactions or the deactivation of CaO under steam atmosphere.

In summary, we infer that the addition of active support materials cannot hinder the release of sulphurous gas from side reactions in both the reduction and oxidation stages; however, the additions can improve the reaction rate. Compared with CaSO_4_/ash and CaSO_4_/cement, the CaSO_4_/clay composite oxygen carrier exhibited excellent attrition resistance and reactivity with steam as the gasifying agent. Hence, the CaSO_4_/clay composite oxygen carrier can be a potential oxygen carrier for coal CLC after being upgraded.

### 3.5 Upgrading the CaSO_4_/clay composite oxygen Carrier using CaO

Thermodynamic analysis and thermogravimetric experiments indicated that the side reaction emitting sulphurous gas was unavoidable, even without the addition of inert materials to CaSO_4_. It seemed that the inhibition of sulphurous gas emission was vital for using CaSO_4_ as an oxygen carrier for coal CLC. Because CaO is deemed as a sulphur adsorption agent in flue gas desulphurization, CaSO_4_/clay was upgraded by adding CaO. CaSO_4_/clay composite oxygen carriers with four mass ratios of CaO to CaSO_4_, *i.e.*, 0.25, 0.5, 1 and 1.25, were prepared. Investigations into the reactivities of the four upgraded CaSO_4_/clay composite oxygen carriers with coal were carried out in a fluidised bed.

**Fig. 9:**
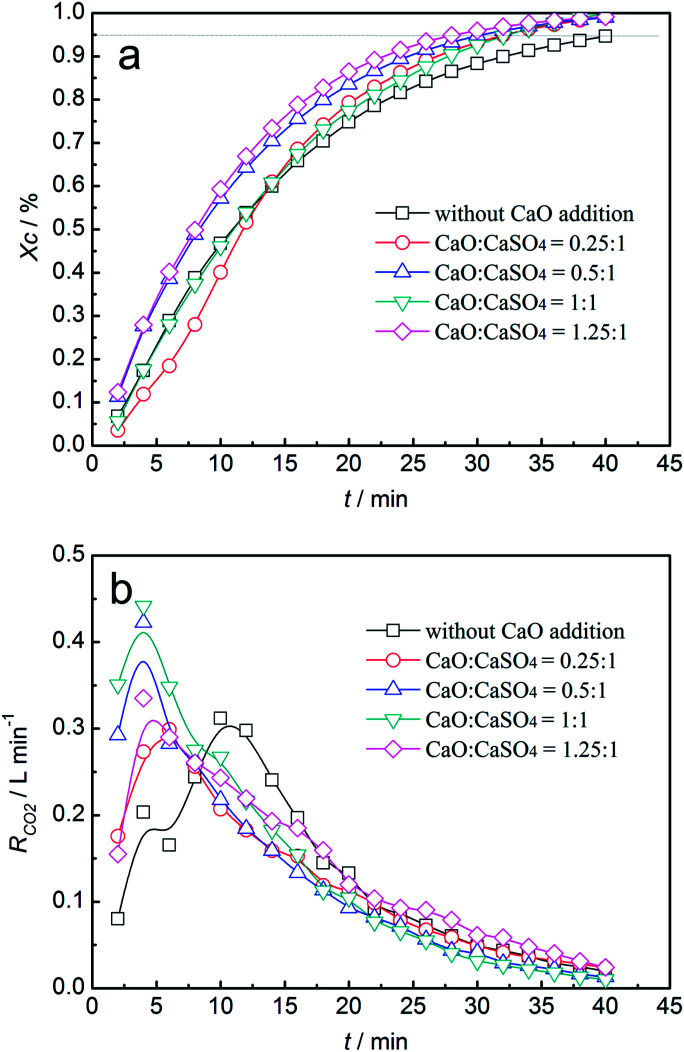
Variation of carbon conversion efficiency (a) and CO_2_ generation rate (b) with time for the CaSO_4_/clay composite oxygen carrier with different CaO additions.

The results for carbon conversion efficiency and CO_2_ generation rate with different CaO additions *versus* time are shown in Fig. 9. As displayed in Fig. 9a, the carbon conversion efficiency increased with the increasing time. Moreover, the carbon conversion efficiency increased with the increasing amounts of CaO. The time to achieve 95% conversion efficiency was shortened from 40 min without the addition of CaO to 28 min with 1.25 mass ratio additions. As was depicted in Fig. 9b, the time of the maximum CO_2_ generation rate advanced with the increasing addition mass ratio. It shortened from 12 min to 5 min. When the mass ratio of CaSO_4_ to CaO was equal to 1, the maximum value of the CO_2_ generation rate was 0.43 L min^−1^. The addition of CaO enhanced the reaction between the CaSO_4_/clay composite oxygen carrier and coal with steam as the gasifying agent.

**Fig. 10:**
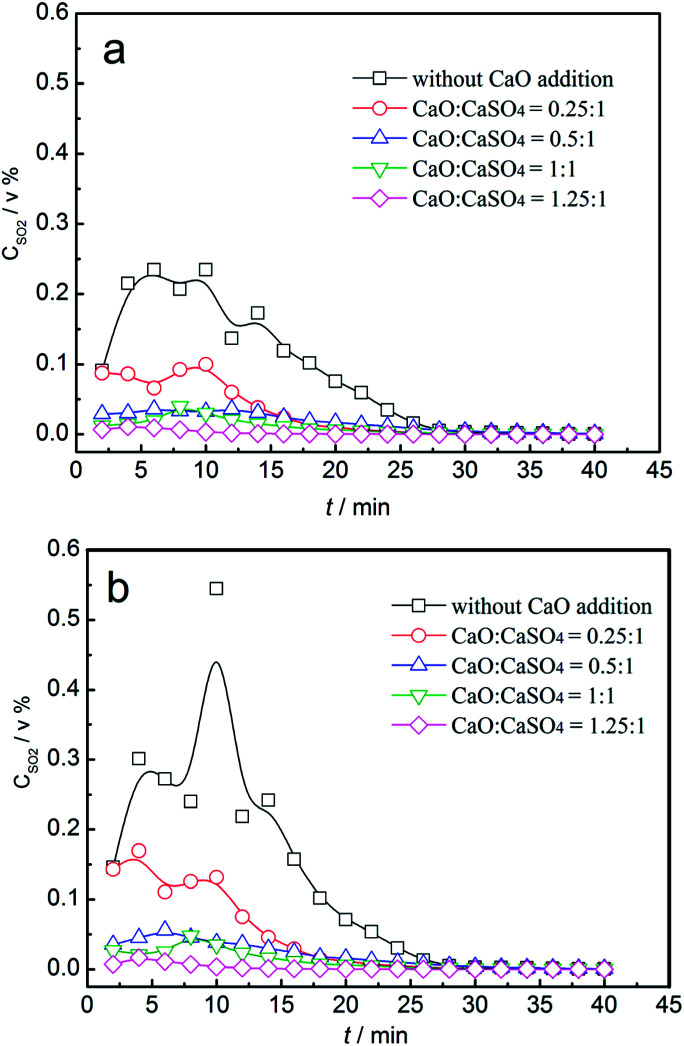
Variation of gas concentrations of SO_2_ with time for the CaSO_4_/clay composite oxygen carrier with different CaO additions; (a) reduction stage, (b) oxidization stage.

The effect of CaO addition on the concentration of sulphurous gas is demonstrated in Fig. 10. In the reduction stage, the SO_2_ volume concentration increased first and then decreased, with the maximum value of 0.24% without the addition of CaO. With the increase in the added amounts of CaO, both the duration of SO_2_ emission and the maximum value of SO_2_ volume concentration decreased. When the mass ratio was higher than 1, the SO_2_ volume concentration was restrained. In the oxidation stage, the SO_2_ volume concentration exhibited a similar trend with that in the reduction stage. The maximum value was 0.54% without the addition of CaO, which revealed that SO_2_ emission during oxidation was significant. With the increasing mass ratio of CaO, both the duration of SO_2_ emission and the maximum value of SO_2_ volume concentration decreased. In summary, SO_2_ release was restrained until the mass ratio of CaO to CaSO_4_ was higher than 1 at both the reduction and oxidation stages. The corresponding oxygen transport capacity of the composite oxygen carriers was about 14.1 wt%.

## 4. Conclusions

From the viewpoint of chemical reaction engineering, the addition of active supports can inhibit the sintering of oxygen carrier particles and enhance the reactivity and structural stability of the oxygen carriers. CaSO_4_ composite oxygen carriers composed of clay, cement, and ash were prepared separately. The following conclusions were obtained: (1) the attrition resistance of CaSO_4_ oxygen carriers composed of clay and cement was improved due to bond action of cement and clay. The reaction rate of CaSO_4_ with coal could be improved by adding active support materials. However, due to side reactions, the regeneration of CaSO_4_ deteriorated. (2) Sulphurous gas generation from three types of CaSO_4_ composite oxygen carriers occurred in both the reduction and oxidation stages. The use of a gasifying agent greatly affected the release behaviour of sulphurous gas. Compared with CaSO_4_/ash and CaSO_4_/cement, the CaSO_4_/clay composite oxygen carrier exhibited excellent reactivity with steam as the gasifying agent. (3) Addition of CaO to CaSO_4_/clay could enhance its reaction rate with coal and restrain the release of SO_2_. The SO_2_ release was restrained until the mass ratio of CaO to CaSO_4_ was higher than 1 in both the reduction and oxidation stages. At this point, the corresponding oxygen transport capacity of composite oxygen carriers was about 14.1 wt%. The addition of a large amount of CaO to the CaSO_4_ oxygen carrier is a good strategy to prevent the release of sulphurous gas.

## Conflicts of interest

There are no conflicts to declare.

## Supplementary Material

RA-008-C8RA03425G-s001
