# Supplementary material for: Characteristics of a CaSO4 composite oxygen carrier supported with an active material for in situ gasification chemical looping combustion of coal
Source: RSC Adv. 2018 Jun 27;8(41):23372–81. doi: 10.1039/c8ra03425g (PMC9081615; doi:10.1039/c8ra03425g)
Supplement: RA-008-C8RA03425G-s001 [file RA-008-C8RA03425G-s001.pdf]

Electronic Supplementary Material (ESI) for RSC Advances.
This journal is © The Royal Society of Chemistry 2018

Supplement material:

Characteristics of CaSO4 Composite Oxygen Carrier Supported with Active Material for In-situ Gasification Chemical Looping Combustion of Coal

Based on the Gibbs free energy minimization method, the thermodynamic analysis of reaction system including coal (simplified as carbon here), $CaSO_4$ and the components of inert materials varying with the temperature was performed using HSC 6.0. The common gaseous species considered in the product is CO (g) and CO_2 (g). In particular, the considered sulfurous species are SO_2 (g) CS_2 (g) and COS (g), where the solid species are CaS, $CaSO_4$, CaO, and $CaCO_3$ mainly. The considered species of the components of inert materials are SiO_2, $CaSiO_3$, Ca_3SiO_5, and $Ca_3Si_2O_7$ for SiO_2, Al_2O_3, $CaAl_2O_4$ and $CaAl_2O_7$ for Al_2O_3, FeS, Fe_2O_3, Fe, FeO, Fe_3O_4, $CaFe_3O_5$, and $CaFe_5O_7$ for Fe_2O_3, respectively. The gasified agent CO_2 and steam leaves out of consideration. In a reaction system, the amount of carbon (substituting for coal), $CaSO_4$ and one component of inert material is set as 2 kmol, 1 kmol and 0.5 kmol, respectively. The mole ratio of $CaSO_4$ to carbon is 0.5, according to $CaSO_4$+2C=CaS+2CO_2. The amount of the inert component is excessive to make the effect of support components on $CaSO_4$ noticeable.

From the XRD patterns of fresh composite oxygen carriers, we can know that the calcination at high temperature seldom make the reaction between $CaSO_4$ and the inert component possible to product the new species. However, the addition of carbon or reductive gases not only can facilitate the decomposition of $CaSO_4$ to CaS or CaO [S1], but also can promote the reaction between $CaSO_4$ and SiO_2, Al_2O_3, or Fe_2O_3 to generate the eutectic mixtures and sulfurous gas [S2], resulting in the deactivation of $CaSO_4$ oxygen carrier. The equilibrium amount of the species varies with the temperature in C-$CaSO_4$ system was depicted in Fig. S1. When the temperature is lower than 950 ^{o}C, the CaS is the main reduction product. The amount of CaO increased slowly with the increasing temperature when the temperature is higher than 950 ^{o}C, which attributes to the reductive decomposition reaction of $CaSO_4$.

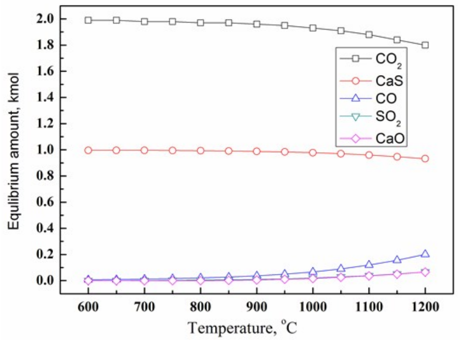

Fig. S1. Equilibrium amount of the species varies with the temperature in C-$CaSO_4$ system

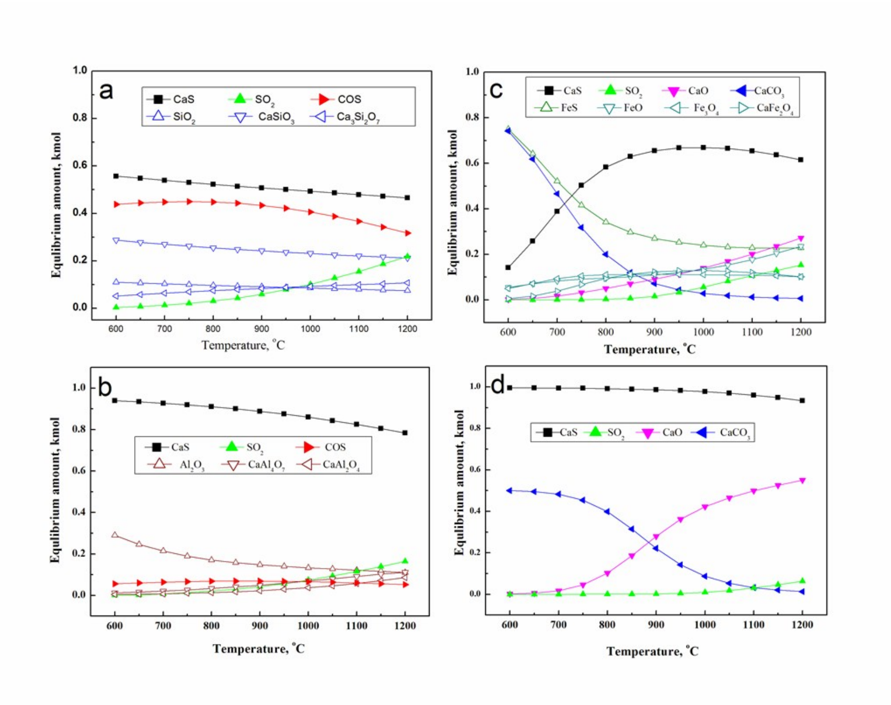

Fig. S2. Effect of the addition of inert components on equilibrium amount of the species varies with the temperature: (a) SiO_2; (b) Al_2O_3; (c) Fe_2O_3; (d) CaO

Equilibrium amount of the species varies with the temperature with the addition of inert components was illustrated in Fig. S2. As Fig. S2a shown, the generation of $CaSiO_3$ and COS with the existence of SiO_2 make lower than 60% $CaSO_4$ generate to the desired product CaS. Meanwhile, the amount of SO_2 begins to increase as the increasing temperature and the decreasing amount of COS when the temperature is higher than 800 ^{o}C. Effect of Al_2O_3 on the C-$CaSO_4$ system was demonstrated in Fig. S2b. Similarly, the amount of the desired product CaS is less than that without the addition of Al_2O_3, due to the generation of $CaAl_3O_7$ and $CaAl_2O_4$. With the increasing temperature, the amount of CaS decreases slowly while the amount of SO_2 increases. Effect of Fe_2O_3 on the C-$CaSO_4$ system was demonstrated in Fig. S2c. Fe_2O_3 itself is a kind of oxygen carrier. However, it seems that FeS is favorable thermodynamically, which does harm to the regeneration of $CaSO_4$. Nevertheless, when the temperature is lower than 850 ^{o}C, with the increasing temperature, the amount of CaS increases while the amount of FeS decreases. In a wide temperature range from 900~1100 ^{o}C, the amount of CaS hardly changes, though the amount of CaO is increasing. Besides, the eutectic mixtures $CaFe_2O_4$ formed. Summarily, the formation of the eutectic mixtures with addition of SiO_2, Al_2O_3, or Fe_2O_3 is detrimental for the regeneration of

$CaSO_4$ oxygen carrier. Moreover, when SiO_2, Al_2O_3 and Fe_2O_3 exist in C-$CaSO_4$ system at the same time, the more complex eutectic mixtures will generate. Different from above three components, CaO is the decomposition side-product of $CaSO_4$ oxygen carrier. As shown in Fig. S2d, the addition of CaO into the C-$CaSO_4$ system can inhibit the generation of SO_2. The emission amount of SO_2 is still small when temperature is higher than 1050 ^{o}C.

References

[S1] Bei Yan, Liping Ma, Jun Ma, Zecheng Zi, Xiaodan Yan. Mechanism Analysis of Ca, S Transformation in Phosphogypsum Decomposition with Fe Catalyst. Ind. Eng. Chem. Res. 2014, 53:7648−7654

[S2] N Mihara, D Kuchar, Y Kojima,H Matsuda. Reductive decomposition of waste gypsum with SiO_2, Al_2O_3, and Fe_2O_3 additives. Journal of Material Cycles & Waste Management, 2007, 9(1):21-26
